# Objective interpretation of intrapartum cardiotocography images using attention-guided convolutional neural networks

**DOI:** 10.3389/fped.2026.1717012

**Published:** 2026-02-18

**Authors:** Xinghe Zhou, Tianxin Qiu, Chunxia Lin, Jun Zhou, Shiling Jiang, Litao Wang, Li Feng, Xinhao Wang, Qingshan You

**Affiliations:** 1 Faculty of Science, Civil Aviation Flight University of China, Chengdu, Sichuan, China; 2 Department of Obstetrics, West China Longquan Hospital Sichuan University, The First People's Hospital of Longquanyi District Chengdu, Chengdu, Sichuan, China

**Keywords:** CBAM, computer vision, deep learning, EfficientNet, electronic fetal monitoring, fetal status classification, intrapartum monitoring

## Abstract

**Objective:**

Automated analysis of Electronic Fetal Monitoring (EFM) is essential for the precise assessment of fetal health. However, the subjective interpretation and expertise-dependent nature of conventional cardiotocogram (CTG) analysis hinder diagnostic consistency. This study aims to develop an objective interpretation approach comprising a systematic preprocessing pipeline for signal reconstruction and an attention-guided convolutional neural network for pattern classification to mitigate the risk of missed diagnoses.

**Methods:**

A computer vision-based deep learning approach was developed. The workflow begins with a systematic preprocessing pipeline, where raw CTG images undergo grid removal, resampling, and curve reconstruction to generate standardized signal inputs. These signals are analyzed by a classifier based on the EfficientNet-B0 architecture, enhanced with a Convolutional Block Attention Module (CBAM). This attention mechanism enables the model to focus on clinically significant morphological features. The model was trained on a private clinical dataset using clinician-labeled FIGO classifications (Normal vs. Suspicious/Abnormal) as the primary outcome. To evaluate its clinical utility and robustness, the model was externally validated on the public CTU-UHB dataset, using objective umbilical artery pH levels (pH ≥7.05 vs. pH <7.05) as the benchmark.

**Results:**

On the internal clinical dataset, the model achieved an accuracy of 92.66% and a macro-average F1-score of 92.14%. When tested on the external CTU-UHB dataset, the model maintained an accuracy of 95.65%. These results indicate that the proposed algorithm aligns with expert visual classification and remains consistent when validated against objective physiological outcomes (pH levels). This consistency across benchmarks supports the potential robustness and clinical relevance of the learned morphological features.

**Conclusion:**

This study presents an objective method for intrapartum CTG analysis. By integrating signal standardization with automated feature learning, the proposed approach addresses the inherent subjectivity of manual interpretation. It serves as a potential clinical decision support tool to assist in the consistency of fetal status assessment.

## 1 Introduction

Electronic Fetal Monitoring (EFM) is a non-invasive diagnostic technique that provides a vital means for assessing fetal health in utero by continuously monitoring key physiological parameters such as fetal heart rate (FHR) and uterine contractions (UC) [1–3]. The ease of operation and capability for prolonged continuous monitoring of EFM has led to its emergence as a routine clinical tool in prenatal care [4]. The fetal heart rate curve is a valuable source of information with regard to the health of the foetus. Clinicians generally undertake the assessment of risks, such as those pertaining to fetal distress, by means of an analysis of the dynamic coupling relationship between FHR and UC. Nevertheless, the visual interpretation of EFM tracings is heavily dependent on clinical expertise, and is subject to significant inter-observer variability and subjectivity [5]. This subjectivity has the potential to result in interpretation errors or overlooked non-reassuring signs, thereby affecting the quality of clinical decision-making [6].

The advent of widespread artificial intelligence (AI) implementation in the domain of medical data analysis has led to a surge of interest in machine learning-based automated EFM analysis, a subject that has become a focal point in research endeavours. Conventional methods generally entail the manual extraction of time-domain and frequency-domain characteristics from FHR signals (e.g., baseline variability, accelerations, decelerations) and their subsequent amalgamation with classifiers such as support vector machines or random forests to evaluate fetal health [7]. While these methodologies demonstrate a certain degree of efficacy, the process of feature engineering is intricate and dependent on expertise, thereby posing a challenge to the balancing of real-time requirements and classification accuracy [8]. In recent years, deep learning techniques have demonstrated significant advantages in the classification and detection of anomalies in FHR signals, due to their powerful hierarchical feature learning capabilities. However, the majority of these methodologies are predicated on one-dimensional time-series signals following a process of preprocessing, and they still cannot completely avoid manual intervention and information loss during the signal preprocessing stage. Presently, research in the domain of AI-based EFM analysis is predominantly categorised into two strands.

Firstly, traditional workflows combine manual feature extraction with machine learning models. Secondly, time-series signal analysis methods are based on deep neural networks (e.g., convolutional neural networks, recurrent neural networks) [9]. It is important to note that the majority of these approaches do not make direct use of the extensive amount of data from fetal heart monitoring images, which are commonly used in clinical practice [10]. Conversely, they are dependent on converted numerical signals, which increases the complexity of data preparation and fails to align with physicians’ actual interpretation workflows and visual diagnostic habits [11].

**Figure 1:**
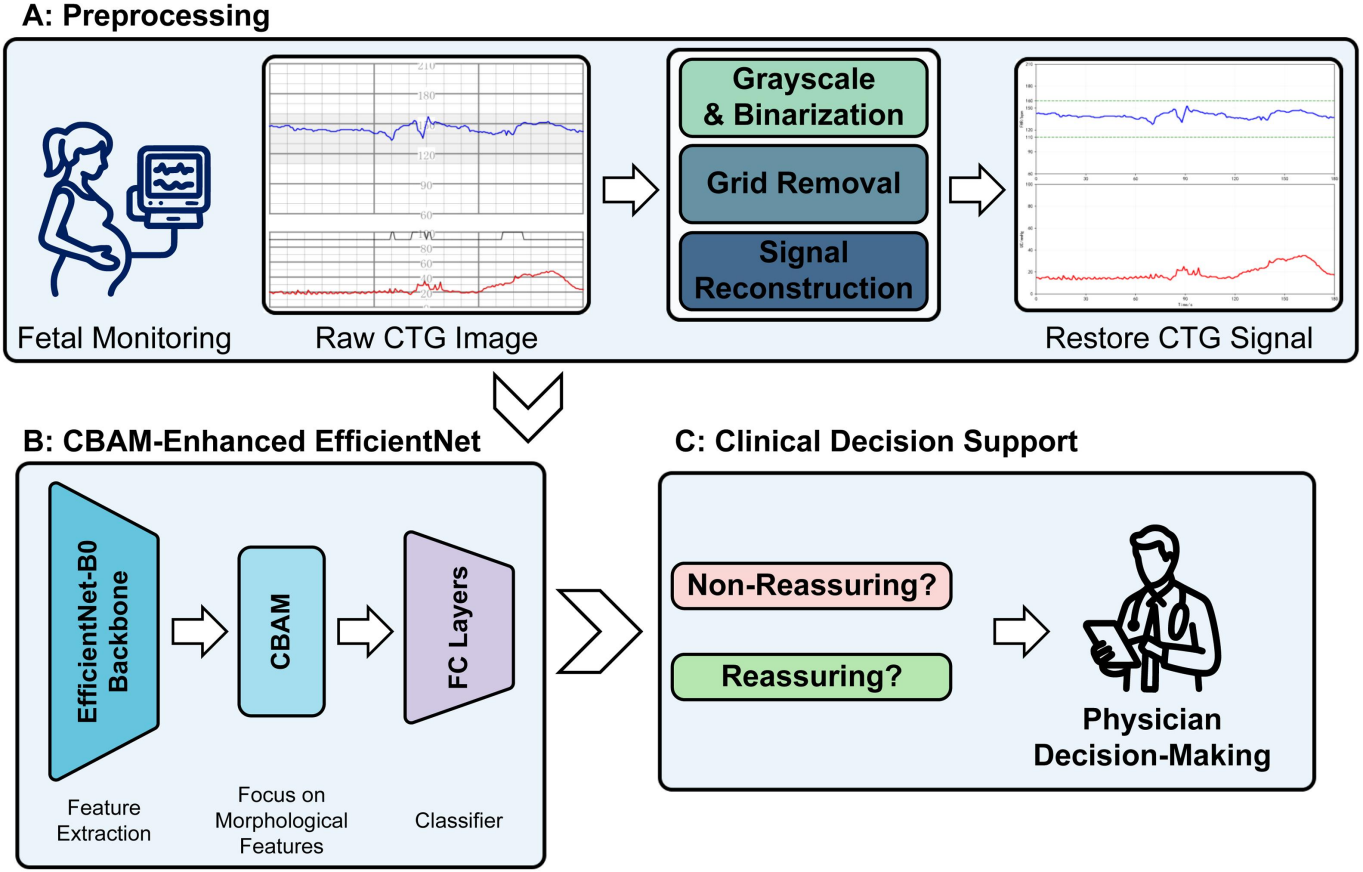
The proposed Framework: a convolutional block attention module (CBAM)-enhanced EfficientNet for cardiotocography (CTG) classification. The workflow consists of three main phases: **(A)** Preprocessing: This phase transforms raw CTG images through a three-step pipeline comprising (1) Grayscale & Binarization, (2) Grid Removal, and (3) Signal Reconstruction to extract clean signal traces. **(B)** CBAM-Enhanced EfficientNet: The reconstructed signals are processed using an EfficientNet-B0 backbone for feature extraction. The integrated CBAM prioritizes morphological features before passing them through Fully Connected (FC) layers. **(C)** Clinical Decision Support: The classifier outputs a prediction (Reassuring vs. Non-Reassuring) to assist physicians in the final decision-making process.

The present paper proposes a computer vision-based approach for fetal heart monitoring image analysis that utilizes deep learning to address these issues. By implementing a systematic preprocessing pipeline to reconstruct standardized signals from raw reports, and subsequently employing a deep learning model for automated feature learning, this approach aims to minimize the subjectivity and reduce the potential information loss inherent in manual signal interpretation and visual diagnosis. As demonstrated in Figure 1, this approach employs a systematic preprocessing pipeline followed by a convolutional neural network with a fusion attention mechanism to learn potentially informative morphological feature representations. The primary contributions of this paper are as follows:

- A fetal heart monitoring image preprocessing workflow is proposed, which is designed to retain essential signal information while minimizing background interference, such as grids and shadows, through signal reconstruction.
- A deep learning method has been designed based on the EfficientNet architecture and the Convolutional Block Attention Module (CBAM) to focus on morphological features associated with potential fetal risks.
- A validation process is conducted across internal and external datasets, demonstrating the method’s performance with an accuracy of 92.66% internally and 95.65% on the CTU-UHB dataset. This method is proposed as a potential clinical support tool to assist in reducing the risk of missed diagnoses.

This method aims to improve the consistency of CTG interpretation by aligning the automated workflow with clinical diagnostic habits. The results suggest that the proposed approach has the potential to provide warnings of fetal risks, serving as a candidate clinical support tool to assist in reducing the risk of missed diagnoses. The subsequent sections are structured as follows: Section 2 provides a detailed account of the materials and methods employed in the study. Section 3 presents the experimental results and analysis. Finally, Section 4 concludes the paper with a discussion and future directions.

## 2 Materials and methods

### 2.1 Research framework

The objective of this study is to develop an objective interpretation approach based on deep learning for the automated analysis of fetal heart rate (FHR) and uterine contraction (UC) signals. This method aims to provide an objective evaluation of fetal status, offering obstetricians a supportive instrument for clinical decision-making intended to help address the variability associated with subjective interpretation. To potentially overcome certain limitations of conventional Electronic Fetal Monitoring (EFM) analysis, the present study proposes an automated deep learning approach [12] designed to be lightweight and suitable for clinical monitoring environments.

As illustrated in Figure 1, the research framework incorporates the following stages:

- Data acquisition: Fetal FHR and maternal UC signals are acquired via electronic fetal monitors, followed by the generation of image-based reports;
- Expert annotation: Raw reports are annotated by domain experts to establish a reliable ground truth for the training dataset;
- Preprocessing: Raw images undergo a systematic pipeline to remove background interference (e.g., grids) and reconstruct standardized signals;
- Feature learning: The reconstructed data is processed by a neural network with a fusion attention mechanism to automatically learn discriminative morphological features;
- Classification: The trained approach is implemented to achieve automated classification of fetal status into Reassuring or Non-Reassuring categories.

In contrast to conventional methodologies that rely on manual feature engineering, the deep learning model in this approach is data-driven. By automatically extracting deep features from digitized signals, the approach aims to reduce the impact of human bias and minimize the potential for information loss often encountered in manual visual diagnosis.

The primary contribution of this study is an automated CTG interpretation approach designed with the goal of supporting efficient operation. By providing timely alerts regarding potential fetal risks, this method may alleviate the need for constant expert oversight and supports clinical consistency. Furthermore, the lightweight design facilitates its deployment in diverse clinical settings, including primary healthcare centers, thereby offering a scalable solution for fetal status assessment.

### 2.2 Datasets

In this study, we adopted a rigorous “Internal Development + External Validation” strategy to evaluate the proposed framework. The primary model development and internal testing were conducted using a large-scale private clinical dataset collected from West China Longquan Hospital Sichuan University (The First People’s Hospital of Longquanyi District Chengdu). To further assess the model’s robustness and generalization capability across different populations and acquisition devices, we utilized the publicly available CTU-UHB dataset as an independent external validation set.

#### 2.2.1 Internal clinical dataset (private dataset)

The core dataset for this study was collected from West China Longquan Hospital Sichuan University (The First People’s Hospital of Longquanyi District Chengdu). It comprises 1,622 high-fidelity CTG recordings collected recently between January 25, 2025, and June 25, 2025. This specific temporal window is intended to capture contemporary obstetric populations and current clinical practices. Each record consists of synchronous FHR and uterine contraction (UC) signals with a standardized duration of 20 min. All recordings were annotated by senior obstetricians based on the FIGO guidelines.

#### 2.2.2 External validation dataset (CTU-UHB)

To verify the cross-center generalization capability of our model, we employed the CTU-UHB intrapartum CTG database [13]. This dataset contains 552 recordings obtained from the Department of Obstetrics and Gynecology at the University Hospital in Brno, Czech Republic. The signals were acquired using scalp electrodes (102 cases), ultrasound probes (412 cases), or a combination of both, sampled at 4 Hz. This dataset serves as a standard benchmark in the field, representing a distinct European population profile compared to our internal Chinese cohort.

#### 2.2.3 Dataset comparison and label definition

To assess the domain shift between the internal training distribution and the external testing distribution, we performed a comprehensive statistical comparison of demographic and clinical characteristics, as summarized in Table 1.

**Table 1:** Demographic and clinical characteristics of the study populations.

Characteristic	Internal Dataset (Private) (n=1,622)	External Dataset (CTU-UHB) (n=552)	P-value
Maternal characteristics			
Maternal Age, mean (SD), years	29.3 (3.2)	29.7 (4.5)	0.054
Nulliparous, No. (%)	1,080 (66.6%)	376 (68.1%)	0.543
Pregnancy and labor data			
Gestational Age, mean (SD), weeks	38.64 (1.6)	40.0 (1.1)	<0.001${}^{*}$
Delivery Mode, No. (%)			<0.001${}^{*,a}$
Vaginal Delivery	845 (52.1%)	506 (91.7%)	
Cesarean Section	777 (47.9%)	46 (8.3%)	
Fetal Status Classification, No. (%)			<0.001${}^{*,b,c}$
Normal	1,020 (62.9%)	508 (92.0%)	
Suspicious	558 (34.4%)	–	
Abnormal	44 (2.7%)	44 (8.0%)	
Reassuring(Normal)	1,020 (62.9%)	508 (92.0%)	
Non-Reassuring(Suspicious/Abnormal)	604 (37.1%)	44 (8.0%)	
Neonatal Outcomes (Ground Truth)			
Umbilical Artery pH, mean (SD)	7.30 (0.06)	7.23 (0.11)	<0.001${}^{*}$
Apgar Score at 5 min <7, No. (%)	36 (2.2%)	19 (3.4%)	0.155
Birth Weight, mean (SD), kg	3.1 (0.4)	3.4 (0.5)	<0.001${}^{*}$
Male Sex, No. (%)	838 (51.7%)	286 (51.8%)	0.992

Note the significant differences in delivery mode and gestational age, indicating distinct clinical profiles between the two cohorts.

Values are presented as mean (SD) for continuous variables or number (%) for categorical variables. The External Dataset statistics were re-calculated from raw signals to ensure accuracy.

Comparison between groups was performed using Welch’s *t*-test for continuous variables and Pearson’s Chi-square test for categorical variables.

${}^{a}$Comparison of the overall distribution of delivery modes.

${}^{b}$Comparison of the distribution of the three categories (Normal, Suspicious, Abnormal).

${}^{c}$Outcome Definition: The Internal Dataset labels are based on clinician-judged FIGO classifications (Visual interpretation), where *Reassuring* corresponds to FIGO Category I, and *Non-Reassuring* aggregates FIGO Category II (Suspicious) and Category III (Abnormal). The External Dataset (CTU-UHB) labels are defined by objective Umbilical Artery pH levels (Physiological: Normal ≥7.05 vs. Abnormal <7.05).

${}^{*}$Indicates statistical significance (p<0.05).

Statistical analysis reveals significant heterogeneity between the two populations. The internal dataset exhibits a significantly higher Cesarean section rate (47.9% vs. 8.0%, p<0.001) and a lower mean gestational age (38.6 vs. 40.0 weeks, p<0.001) compared to the external CTU-UHB dataset. These discrepancies statistically confirm that the internal dataset represents a distinct clinical population typical of a Tertiary Class A hospital, whereas the external dataset reflects a different demographic profile. The ability of the model to maintain performance on the external dataset, despite these significant statistical differences, provides an assessment of its potential generalization capability.

Regarding the classification labels, a distinct distribution of FHR patterns was observed in the primary internal dataset. As shown in Table 1, a substantial proportion of internal recordings were classified as “Suspicious” (FIGO Category II, 34.4%, n=558). This distribution reflects the clinical reality of a comprehensive Tertiary Class A hospital, where the management of complex pregnancies is routine. Unlike the CTU-UHB dataset which represents historical data, our private dataset comprises recent recordings, ensuring the model is attuned to current obstetric diagnostic complexities.

In this study, we amalgamated the “Suspicious” (Category II) and “Abnormal” (Category III) classes into a single “Non-Reassuring” category. This binary classification strategy (Reassuring vs. Non-Reassuring) is driven by three critical clinical considerations:

- Alignment with clinical triage workflows: In intrapartum monitoring, the primary decision is dichotomous—identifying whether a fetus is “Reassuring” (requiring routine care) or “Non-Reassuring” (requiring heightened surveillance or intervention). Both FIGO Category II and Category III warrant clinical attention, functionally distinguishing them from the “Normal” Category I in a screening context.
- Physiological heterogeneity: Fetal tolerance to stress varies significantly due to individual placental reserve. A morphological pattern classified as “Suspicious” in a vulnerable fetus may rapidly progress to acidosis, whereas the same pattern might be tolerated by a healthy fetus. To address potential missed findings, our labeling strategy emphasizes a sensitive identification of morphological patterns, flagging any trace with potential risk characteristics.
- Risk stratification (minimizing false negatives): As a clinical decision support system designed for screening, the cost of a false negative (missing a fetus in distress) far outweighs a false positive. Amalgamating these classes biases the model towards high sensitivity, with the aim of supporting clinicians as a supplementary tool during fetal monitoring.

### 2.3 Data preprocessing

**Figure 2:**
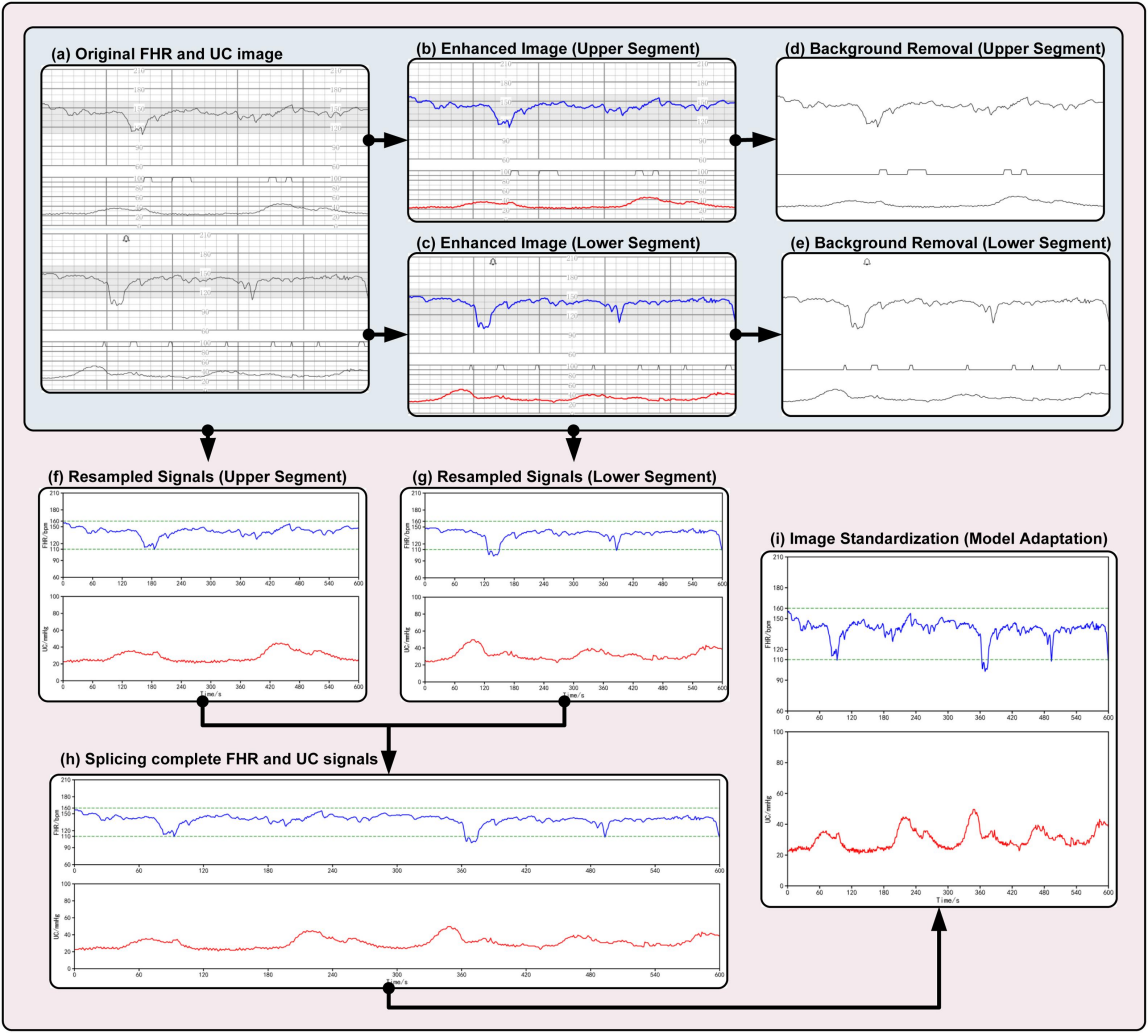
Detailed visualization of the preprocessing pipeline. The raw CTG images undergo a multi-stage transformation to ensure high-quality model input: **(a)** Original raw image containing both FHR (top) and UC (bottom) signals with background grids; **(b,c)** Separation and enhancement of the upper (FHR) and lower (UC) signal regions; **(d,e)** Background removal using adaptive thresholding and morphological operations to eliminate grid lines; **(f,g)** Resampling of the extracted signals to correct temporal alignment; **(h)** Splicing of the segmented signals to reconstruct the complete 20-min recording; **(i)** Final image standardization (Z-score normalization) to generate the model-ready input tensor. This rigorous process ensures that the deep learning model focuses on physiological patterns rather than noise artifacts.

As demonstrated in Figure 2, raw fetal heart monitoring images obtained from hospitals include grid lines and prominent shadowed areas that assist physicians in their observations. In the event of such images being input directly into a neural network for the purpose of feature extraction, the underlying network could potentially capture features irrelevant to the diagnosis process. These features may include grids and shadows, with the result that clinically significant fetal heart rate and uterine contraction curves may be overlooked. Consequently, it is advantageous to remove this superfluous information to allow the network to focus on clinically relevant patterns. This approach is designed to help the network focus on feature images with diagnostic relevance, which may contribute to improved learning efficiency and robustness.

#### 2.3.1 Background mesh removal

**Figure 3:**
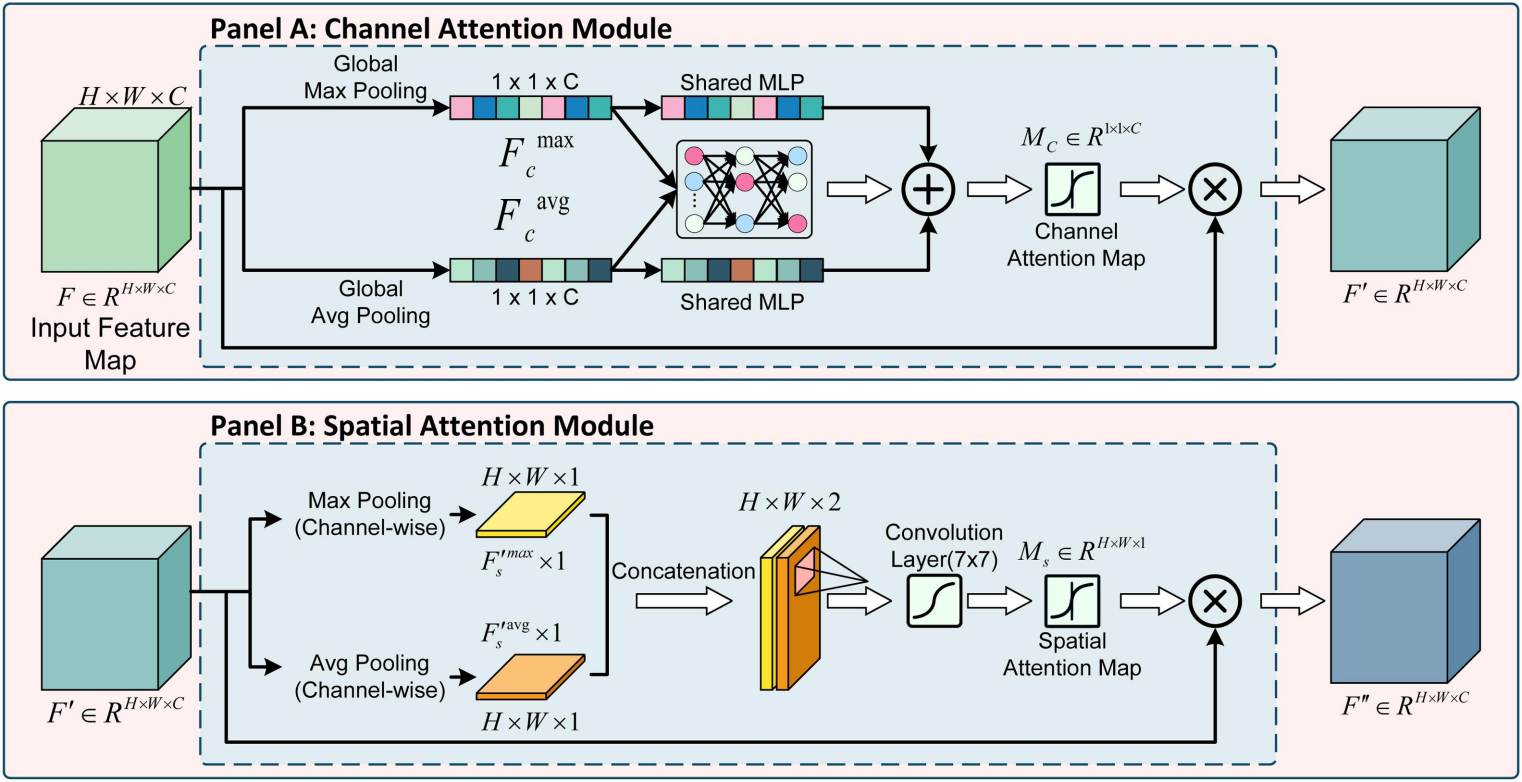
Architecture of the Convolutional Block Attention Module (CBAM). CBAM sequentially infers attention maps along channel and spatial dimensions to refine features. **(A)** Channel Attention Module: Aggregates spatial information to determine “what” to emphasize, using a shared MLP to produce $M_{c}$. **(B)** Spatial Attention Module: Aggregates channel information to determine “where” informative features lie, using a 7×7 convolution to produce $M_{s}$. The final feature $F^{\prime \prime }$ is obtained by serial refinement: $F^{\prime \prime }=M_{s}⊗(M_{c}⊗F)$.

In images obtained for the purpose of fetal heart monitoring, clinically significant fetal heart curves and uterine contraction curves are typically easily identifiable due to their higher pixel intensity. The following preprocessing workflow is employed on the basis of this characteristic: initially, the raw image is converted to grayscale. In grayscale space, the background grid and shadow areas exhibit lower pixel values. Therefore, the implementation of a global threshold (set to 65 in this study) effectively results in the filtration of background pixels with low grayscale values (below the threshold), whilst preserving high-grayscale pixels that represent the target curves. Following the implementation of the binarization process, the resulting image reduces background interference while retaining the key physiological signals (fetal heart rate curve and uterine contraction curve), as illustrated in Figure 3.

#### 2.3.2 Resampling

The objective of this study is to extract clinically meaningful digitized fetal heart rate (FHR) and uterine contraction (UC) curve data from preprocessed binary images. To this end, a coordinate-mapping-based resampling method has been employed. The fundamental premise of this methodology is to establish a linear mapping relationship between the image pixel space and the physiological signal value range, as delineated by the following specific workflow:

Firstly, the range of physiological signals along the vertical axis of the image must be determined. The FHR ranges from 60 to 210 bpm, and the UC ranges from 0 to 100 mmHg. In view of the aforementioned considerations, it is recommended that a linear mapping model [14] be established between the vertical coordinate (y) of pixels in the image and their corresponding physical values (v):

$$v=v_{\min}+\frac{\left(y-y_{\min}\right)}{h}\times \left(v_{\max}-v_{\min}\right)$$ (1)

In this context, $v_{\min}$ and $v_{\max}$ denote the lower and upper limits of the signal range, respectively; h signifies the height of the image’s valid region; and $y_{\min}$ designates the starting vertical coordinate of the valid region.

Subsequently, pixels along the curve are sampled at fixed intervals horizontally (along the time axis). The sampling interval is determined by the total image width and the total clinical duration of the signal recording. The process entails the conversion of the horizontal coordinate of each sampling point into a time series, ensuring consistent temporal resolution. The application of Equation 1 point-by-point for mapping results in the reconstruction of the FHR and UC numerical sequences with temporal resolution matching that of the original.

This method is intended to minimize potential signal distortion caused by image warping, scaling, or non-uniform coordinate systems, supporting the fidelity of the reconstructed numerical data.

#### 2.3.3 Reconstruction and integration of fetal heart monitoring signals

As demonstrated in Figure 2, a comprehensive clinical fetal heart rate monitoring image comprises four distinct curve regions: the upper two curves represent the fetal heart rate (FHR) and uterine contraction pressure (UC) curves recorded during the initial ten minutes, while the lower two curves correspond to the monitoring data from the subsequent ten minutes. In order to extract high-quality digitized signals from the image for use in deep learning models, this study employs the following standardized reconstruction process:

Initially, each ten-minute curve segment undergoes resampling. The pixel positions in images are converted into numerical sequences according to a pre-established coordinate-to-physical-quantity mapping. The FHR signal range is defined as 60–210 bpm, and the UC signal range as 0–100 mmHg.

Consequently, successive signal segments were then concatenated and integrated chronologically to reconstruct complete, background-free 20-min FHR and UC numerical sequences. This process effectively mitigates potential temporal misalignment or amplitude distortion caused by segmented image acquisition, ensuring signal temporal consistency and clinical interpretability.

Finally, to generate the model-ready input tensor, the reconstructed time-series data is mapped to a standardized spatial resolution of 224×224 pixels. Although the raw CTG signal is inherently rectangular along the time axis, this standardization is justified by three technical and empirical considerations:

- Transfer learning compatibility: The EfficientNet-B0 backbone is pre-trained on ImageNet (square inputs). Aligning our input dimensions allows the model to effectively leverage pre-trained low-level visual features (e.g., edges and textures), which is critical for preventing overfitting on medical datasets with limited samples.
- Visual robustness of CNNs: As a visual model, the CNN excels at extracting local morphological features (e.g., the slope of late decelerations or the amplitude of variability) rather than relying on global aspect ratios. This resizing acts as a linear coordinate transformation that preserves the topological structure and relative shape characteristics of the signal curves, maintaining the diagnostic “visual signature”.
- Empirical validation: The validity of this preprocessing strategy is empirically supported by our rigorous 5-fold cross-validation results (Accuracy: 92.66%), which suggest that the standardization process retains key diagnostic features sufficient for high-accuracy classification.

This structured approach provides an accurate and stable data foundation for subsequent automated classification of fetal status.

### 2.4 Network architecture

#### 2.4.1 EfficientNet

EfficientNet is a convolutional neural network architecture that employs a composite scaling approach to simultaneously optimize network depth, width, and input resolution [15]. Unlike traditional scaling methods that focus on a single dimension, this strategy coordinates the expansion of all three dimensions aiming to balance predictive performance with computational efficiency. The architecture series utilizes EfficientNet-B0, derived via neural architecture search, as the baseline. Variants from B1 to B7 are generated using a composite coefficient ϕ to accommodate different computational resources.

The fundamental building block of EfficientNet is the Mobile Inverted Bottleneck Convolution (MBConv) module, which integrates depthwise separable convolution with Squeeze-and-Excitation (SE) attention. This fusion is designed to enrich feature representation with fewer parameters. Specifically, depthwise separable convolution minimizes computational complexity, while the SE module adaptively recalibrates channel weights to prioritize informative features.

The mathematical formulation of the composite scaling method is expressed as Equation (2):

$$d=\alpha^{\phi },\;w=\beta^{\phi },\;r=\gamma^{\phi }$$ (2)

where d,w,r denote the scaling factors for network depth, width, and resolution, respectively; α,β,γ are constant coefficients determined by a grid search, and ϕ is a user-defined coefficient that controls available resources.

Due to the relatively modest scale of the fetal heart monitoring dataset in this study, the EfficientNet-B0 architecture was selected as the feature extraction backbone. This choice aims to control model complexity and prevent overfitting while leveraging robust feature representations pre-trained on the ImageNet dataset. By transferring these learned features to the specific domain of CTG image analysis, the method establishes a reliable foundation for classification despite limited clinical data.

#### 2.4.2 Mobile bottleneck convolution module (MBConv)

The Mobile Inverted Bottleneck (MBConv) module is the fundamental computational unit of the EfficientNet architecture used in this study. This module integrates depthwise separable convolution [16], inverted residual connections [17], and Squeeze-and-Excitation (SE) attention mechanisms to achieve efficient feature representation with reduced computational overhead.

The workflow of an MBConv module begins with a 1×1 convolution to expand the input channel count, followed by a depthwise separable convolution for spatial feature extraction. This operation decomposes a standard convolution into depthwise and pointwise steps, expressed as Equation (3):

DSC(X)=PointwiseConv(DepthwiseConv(X)) (3)

where X denotes the input feature tensor and DSC represents the Depthwise Separable Convolution operation.

To further enhance feature expression, the module incorporates an SE submodule that adaptively recalibrates channel-wise feature responses by generating attention weights through global pooling and fully connected layers, as shown in Equation (4):

$$M_{c}=\sigma \left(W_{2}\cdot \delta \left(W_{1}\cdot \mathrm{GAP}(F)\right)\right)$$ (4)

where F represents the intermediate feature map; GAP⁡(⋅) denotes the Global Average Pooling operator; $W_{1}$ and $W_{2}$ are the weight matrices of the fully connected layers; δ and σ represent the ReLU and Sigmoid activation functions, respectively.

Finally, the module utilizes 1×1 convolutions to project the channels back to the target dimension. These features are then integrated with the original input through an inverted residual connection (skip connection) to facilitate gradient flow, which is defined as Equation (5):

Y=X+F(X) (5)

where Y denotes the output tensor and F(⋅) represents the sequence of transformations within the MBConv module. This architecture is designed to capture characteristic patterns in CTG signals with relatively low computational overhead.

#### 2.4.3 Convolutional block attention module (CBAM)

The Convolutional Block Attention Module (CBAM) is integrated into the EfficientNet backbone to allow the model to selectively prioritize regions within CTG image reports. CBAM is a lightweight, plug-and-play attention module that derives attention weights sequentially across both channel and spatial dimensions, facilitating the network in weighting informative features relative to background information [18].

The module comprises two serial submodules: the Channel Attention Module and the Spatial Attention Module. As illustrated in Figure 3, the Channel Attention Module exploits inter-channel relationships by aggregating spatial information through global average pooling and max pooling. These operations generate a channel attention map $M_{c}\in R^{1\times 1\times C}$, calculated as Equation (6):

$$M_{c}=\sigma (\mathrm{MLP}(\mathrm{AvgPool}(F))+\mathrm{MLP}(\mathrm{MaxPool}(F)))$$ (6)

where $F\in R^{H\times W\times C}$ denotes the input feature tensor; MLP represents a shared multi-layer perceptron, and σ denotes the sigmoid activation function. This map is utilized to recalibrate the importance of each channel.

Subsequently, the Spatial Attention Module determines the focus areas within the feature map, generating a spatial attention map $M_{s}\in R^{H\times W\times 1}$, defined as Equation (7):

$$M_{s}=\sigma \left(\;f^{7\times 7}([\mathrm{AvgPool}(F^{\prime });\mathrm{MaxPool}(F^{\prime })])\right)$$ (7)

where $F^{\prime }$ is the channel-refined feature; [⋅;⋅] denotes the concatenation operation, and $f^{7\times 7}$ represents a convolution operation with a 7×7 kernel.

The final refined feature tensor $F^{\prime \prime }$ is obtained through a two-stage refinement process, as expressed in Equation (8):

$$F^{\prime }=M_{c}⊗F,\;F^{\prime \prime }=M_{s}⊗F^{\prime }$$ (8)

where ⊗ denotes element-wise multiplication. By integrating CBAM, the approach is intended to assign higher weights to potential regions of interest—such as accelerations or decelerations in FHR waveforms—while maintaining robustness against grid noise and background interference.

#### 2.4.4 CBAM-enhanced EfficientNet approach

**Figure 4:**
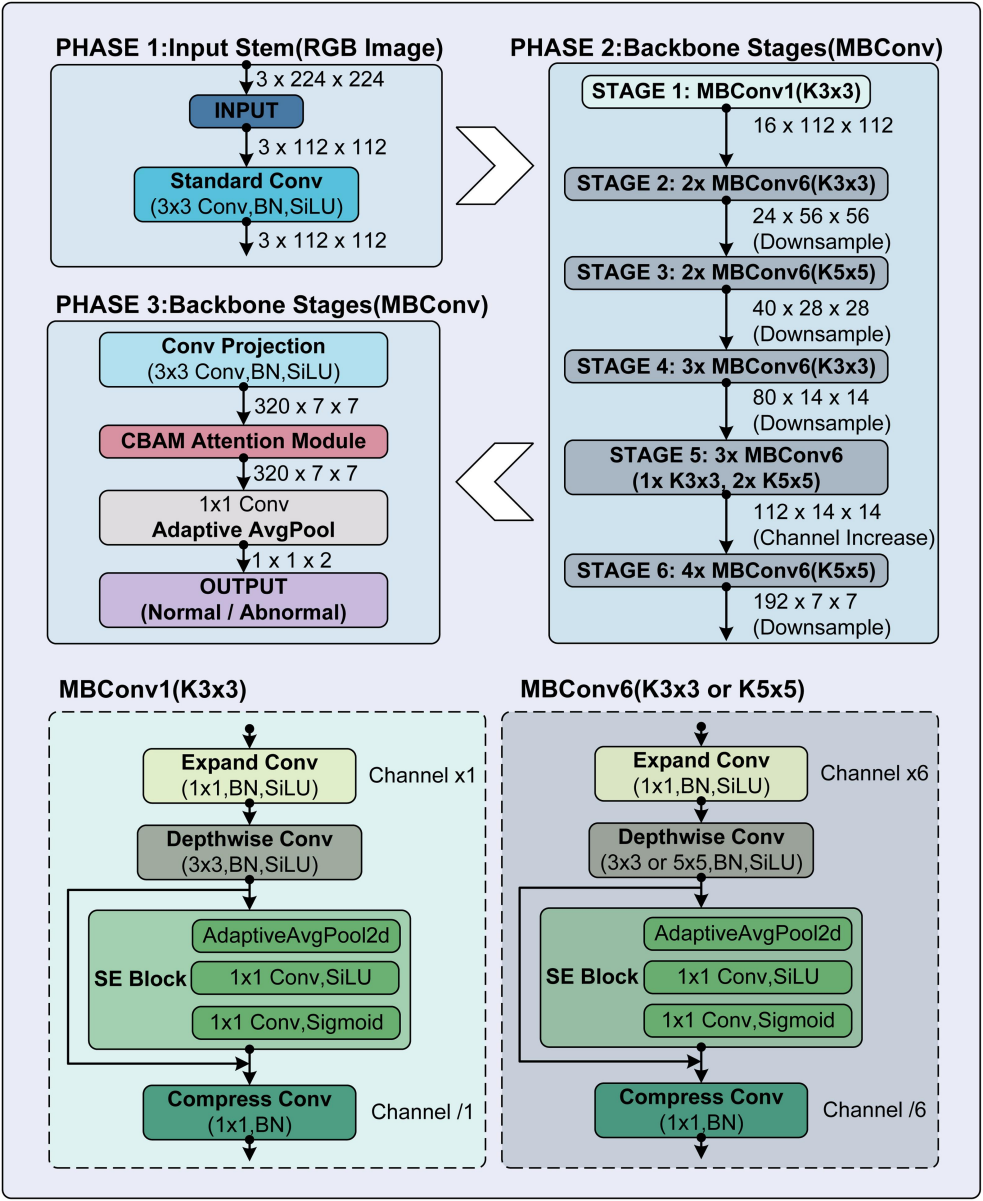
Architecture of the proposed CBAM-enhanced EfficientNet approach. The network pipeline is structured into three primary phases: **(1)** Input Stem: The model accepts a 3×224×224 RGB image (standardized to the pre-trained EfficientNet backbone) and performs initial feature mapping via a 3×3 convolution with Batch Normalization (BN) and Sigmoid Linear Unit (SiLU) to generate a 32×112×112 feature map. **(2)** Backbone Stages: Feature extraction is conducted through six stages of stacked Mobile Inverted Bottleneck Convolution (MBConv) blocks. *MBConv1* (Stage 1) utilizes a 3×3 kernel with a unity expansion ratio, while *MBConv6* (Stages 2–6) expands channels by a factor of 6 and employs varying kernel sizes to capture multi-scale patterns. **(3)** Attention & Output Head: A CBAM block is integrated after the final projection layer (320×7×7) to refine feature weights. The refined features are processed via Adaptive Average Pooling and a fully connected linear layer to output the final classification (Reassuring vs. Non-Reassuring).

To enhance the automated classification of fetal status from CTG image reports, this study proposes a method integrating the EfficientNet-B0 backbone with the Convolutional Block Attention Module (CBAM), the detailed architecture of which is illustrated in Figure 4. By leveraging the composite scaling of EfficientNet-B0, the approach extracts robust hierarchical features from reconstructed signals while maintaining computational efficiency suitable for clinical environments.

The integration of CBAM after the final backbone stage allows the model to adaptively prioritize the most discriminative regions—specifically key morphological patterns in fetal heart rate and uterine contraction curves. By sequentially recalibrating feature representations along both channel and spatial dimensions, this serial attention mechanism aims to improve the detection of morphological variations, such as late decelerations or reduced variability, with minimal additional computational overhead.

### 2.5 Model evaluation metrics

To provide a comprehensive evaluation of the performance of the proposed neural network model in fetal status classification, this study employs several widely recognised evaluation metrics, including accuracy, precision, recall, and F1-score [19]. These metrics provide quantitative assessments from multiple perspectives, including classification accuracy, reliability of positive class discrimination, minority class recognition capability, and overall model discrimination performance. This study comprehensively calculates the aforementioned metrics based on the test set to assess the statistical reliability of the evaluation results. The final results are reported as mean ± standard deviation to illustrate the distribution and variability of the model’s performance [20].

## 3 Experiments and results

### 3.1 Experimental settings

The proposed method was implemented using the PyTorch deep learning framework, with auxiliary data processing performed via NumPy and Scikit-learn. Training and evaluation were conducted on a workstation equipped with an NVIDIA GeForce RTX 4,070 GPU (8 GB VRAM).

Model parameters were optimized using the Adam optimizer with an initial learning rate of $1\times 10^{-4}$ and a batch size of 64. To prevent overfitting, a weight decay of $1\times 10^{-5}$ and an early stopping strategy with a patience of 5 epochs were applied. All input images were uniformly resized to 224×224 pixels and standardized prior to feature extraction.

The computational environment was configured with Python 3.12 and CUDA 12.9 to ensure consistent acceleration and reproducibility across all experimental runs.

### 3.2 Results analysis

To evaluate the performance of the proposed model, this study has designed two sets of control experiments. The initial set of experiments pertains to internal ablation, while the subsequent set focuses on external generalization capability verification.

The objective of the internal ablation experiments was to evaluate the contribution of the Convolutional Block Attention Module (CBAM) to the model’s performance. A comparison was made between the baseline model EfficientNet-B0 and its variant incorporating the CBAM module (hereinafter referred to as CBAM-Enhanced EfficientNet) on the same dataset.

As demonstrated in Table 2, the incorporation of the CBAM module resulted in an improvement in the model’s performance. The test accuracy increased from 92.05% to 92.66%, and the F1-score rose from 91.48% to 92.14%.

**Table 2:** Ablation study results (5-fold cross-validation).

Performance metric	EfficientNet-B0 (Baseline)	CBAM-Enhanced EfficientNet (Ours)	Improvement
Accuracy (%)	92.05 ± 2.14	92.66 ± 2.26	+0.61
Sensitivity (Recall) (%)	89.88 ± 5.11	90.87 ± 5.61	+0.99
Specificity (%)	93.33 ± 1.47	93.73 ± 0.72	+0.40
F1-Score (%)	91.48 ± 2.37	92.14 ± 2.50	+0.66
AUC (%)	97.38 ± 0.98	97.34 ± 1.22	Comparable

Comparison between the baseline EfficientNet-B0 and the proposed CBAM-Enhanced EfficientNet on the private dataset. Values are presented as Mean ± Standard Deviation. Note that while the AUC remains comparable, the inclusion of CBAM shows an increase in Sensitivity, supporting a reduction in potential missed diagnoses.

**Figure 5:**
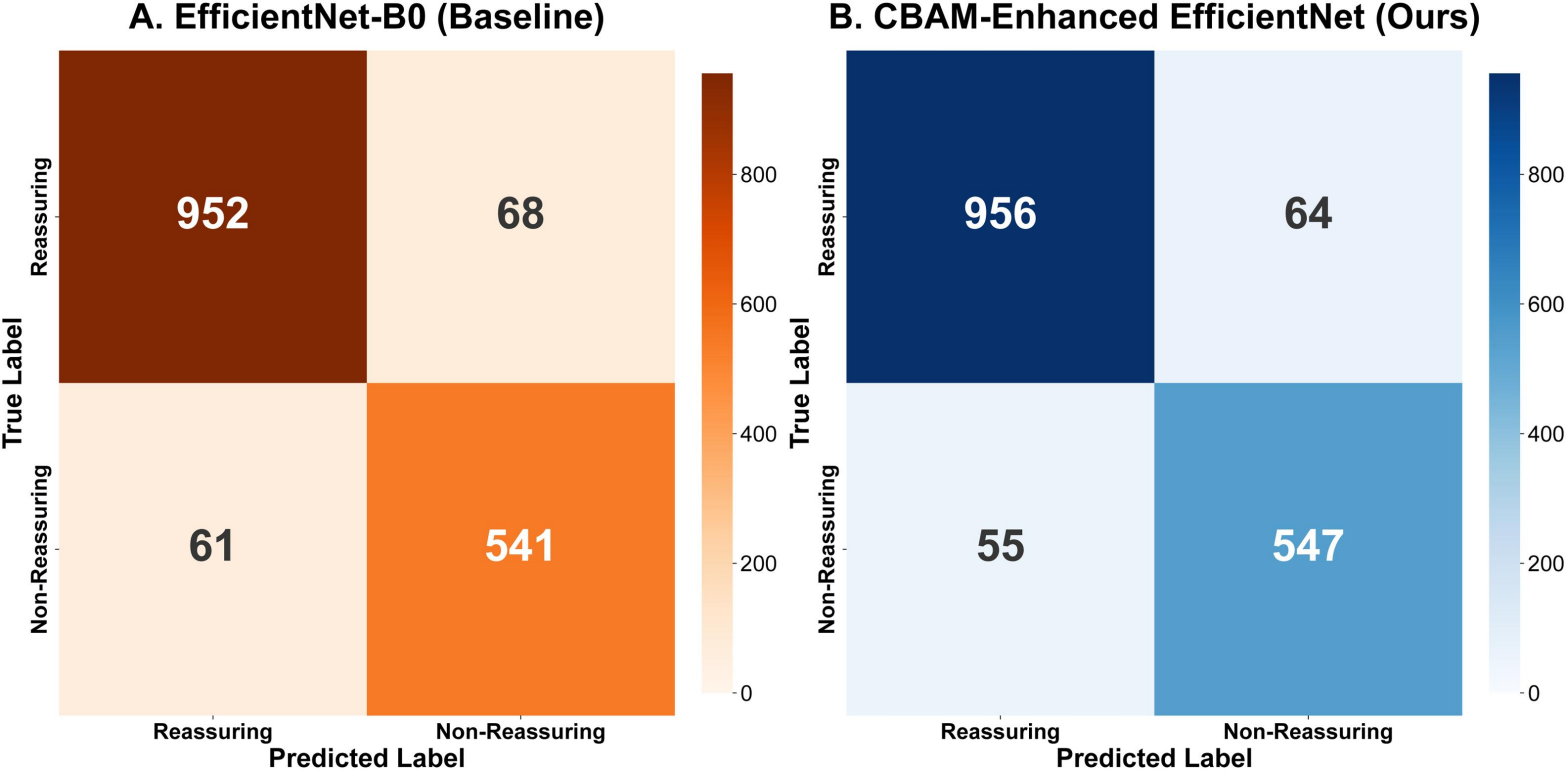
Comparison of aggregated confusion matrices (5-fold cross-validation). **(A)** The Baseline EfficientNet-B0 model. The matrix shows 61 false negatives and 68 false positives. **(B)** The proposed CBAM-Enhanced EfficientNet. By integrating the dual-attention mechanism to recalibrate feature responses, the enhanced model recorded 55 false negatives and 64 false positives. The lower count of false negatives reflects a higher sensitivity in identifying non-reassuring patterns relative to the baseline.

It is noteworthy that the model’s capacity to detect abnormal categories exhibited an observable increase. As visually demonstrated in Figure 5, the proposed CBAM-Enhanced EfficientNet specifically reduced the number of False Negatives (missed diagnoses) from 61 to 55 compared to the baseline. This finding suggests that the CBAM module contributes to the model’s capacity to identify potentially informative morphological patterns. Consequently, the sensitivity for the abnormal category exhibited a targeted improvement. These results suggest that the CBAM module supports the model’s classification performance through adaptive feature calibration, which may assist in addressing the clinical priority of reducing missed diagnoses.

**Figure 6:**
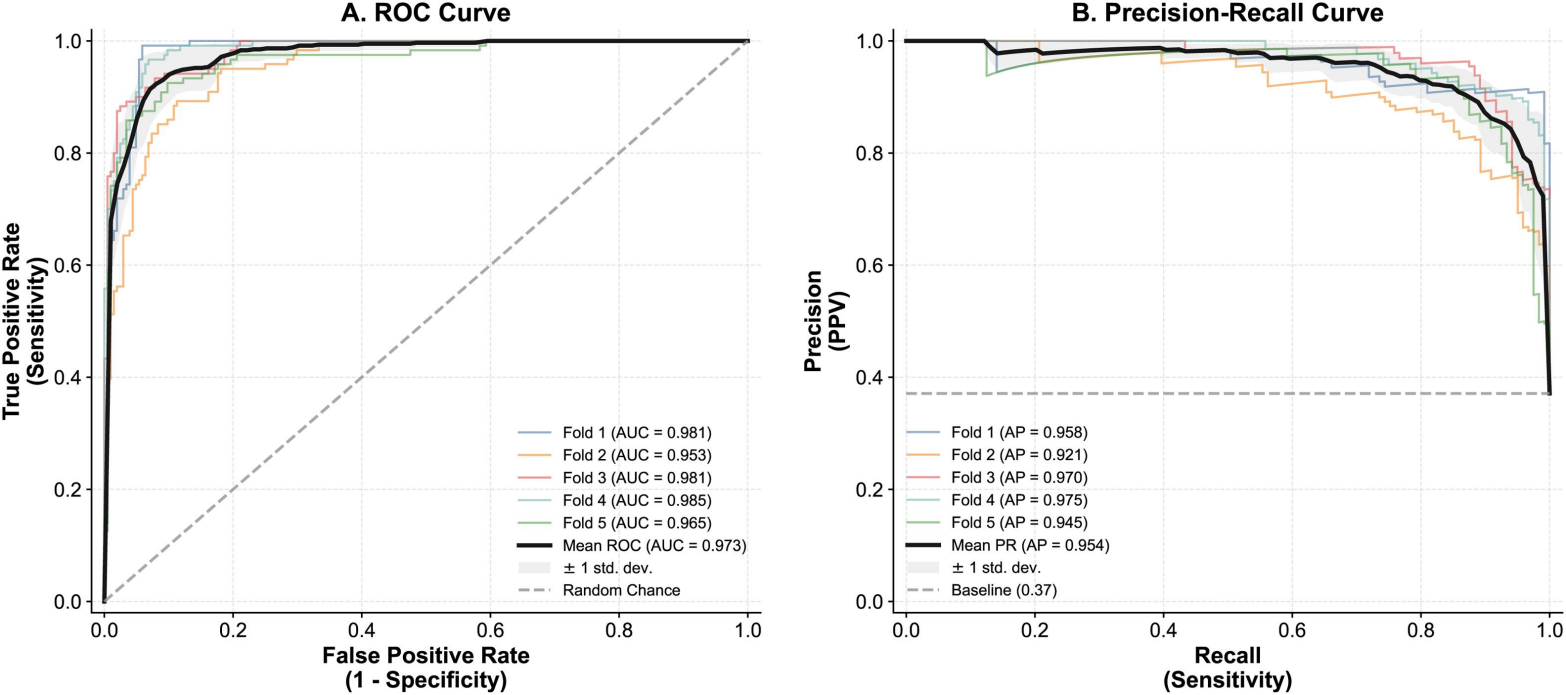
Comprehensive performance evaluation using 5-fold cross-validation. **(A)** Receiver Operating Characteristic (ROC) Curve: The model achieves a high mean AUC of 0.973, suggesting a high degree of separability between Reassuring and Non-Reassuring patterns. The shaded area represents the standard deviation (±1 std), illustrating the consistency of results across folds. **(B)** Precision-Recall (PR) Curve: Designed to assess performance under class imbalance. The high mean Average Precision (AP) of 0.954 is substantially above the random baseline (dashed line, AP = 0.37), indicating that the classification accuracy is supported by sensitivity to the minority (abnormal) class.

To further evaluate the model’s discriminative capability and robustness, particularly in the context of class imbalance, we conducted a comprehensive threshold-independent analysis. As illustrated in Figure 6A, the proposed CBAM-Enhanced EfficientNet achieved a mean Area Under the ROC Curve (AUC) of **0.973** across the 5-fold cross-validation, showing a measured consistency in diagnostic performance across the evaluated metrics. Furthermore, addressing the dataset’s skewed distribution (Reassuring > Non-Reassuring), the Precision-Recall (PR) curve analysis (Figure 6B) yielded a mean Average Precision (AP) of 0.954. This performance is higher than the random baseline (0.37), suggesting that the model’s accuracy is supported by its ability to identify the pathological class to the pathological class rather than a bias toward the majority class.

The validation experiments that were conducted in order to assess the external generalization capability of the proposed CBAM-Enhanced EfficientNet employed the CTU-UHB international public dataset, whilst a comparison of the model’s performance with that of extant methods as reported in the literature was also undertaken. As demonstrated in Table 3, the proposed model attains an accuracy of 95.65% on the CTU-UHB dataset, thereby achieving a higher accuracy compared to the results reported by O’Sullivan et al. Specifically, in comparison with conventional machine learning-based methodologies (e.g., the multi-classifier ensemble employed by Spilka et al., yielding an F1 score of 71.50%; LS-SVM by Georgoulas et al., AUC 68.54%), our deep learning model shows higher performance metrics within this comparison. When evaluated in comparison to contemporary deep learning methodologies (for instance, the CNN-BiLSTM+Attention architecture proposed by Liu et al., achieving an accuracy of 71.71 ± 8.61%), our model recorded a higher accuracy than the CNN-BiLSTM+Attention architecture. These results provide an assessment of the model’s generalization capability on the independent test set.

**Table 3:** Performance comparison with state-of-the-art methods.

Reference	Classifier	Dataset	Samples	Performance metrics (%)		
				Acc	AUC	F1
Krupa et al. [21]	SVM	Private	90	87.00	–	–
Fanelli et al. [22]	ST	Private	122	–	75.00	–
Dash et al. [23]	GM, NB	Private	83	–	–	69.00
Stylios et al. [24]	LS-SVM	CTU-UHB	552	–	72.81	–
Georgoulas et al. [25]	LS-SVM	CTU-UHB	552	–	68.54	–
Comert et al. [26]	EMD+DWT+SVM	CTU-UHB	552	–	64.64	–
O’Sullivan et al. [27]	ARMA+SVM	CTU-UHB	552	83.30	–	–
Liu et al. [28]	CNN-BiLSTM+Attn	CTU-UHB	552	71.71	–	–
Singh et al. [29]	HoloViz+CNN	CTU-UHB	552	69.60	–	–
Ben Barek et al. [30]	DeepCTG (CNN)	CTU-UHB	552	–	74.00	–
Lin et al. [31]	LARA	Private	114	–	87.20	–
Park et al. [32]	SE-ResNet50	Private	22,522	–	88.00	–
Proposed method	CBAM-EfficientNet	CTU-UHB	552	95.65	98.82	87.26
		Private	1,622	92.66	97.34	92.14

Our framework outperforms existing works on the standard public benchmark (CTU-UHB) and demonstrates superior performance on the large-scale private dataset. (“–” indicates the metric was not reported in the original study).

It is noteworthy that the model showed consistent results while maintaining its performance level. In comparison with alternative methodologies—for example, the logistic regression approach employed by Ben Barek et al., which yielded an AUC of 74.0%—our deep learning automated feature extraction method aims to reduce potential information loss associated with manual feature engineering. By directly learning hierarchical morphological patterns from the digitized signals, this method may help address the variability associated with manual visual interpretation associated with manual visual interpretation. Furthermore, the model’s high performance on the CTU-UHB dataset (95.65% accuracy) is consistent with its performance on private datasets (92.66% accuracy), which supports the consistency of the proposed method. However, the robustness of the model across centers and populations necessitates further validation in future research using large-scale, multi-center datasets.

Recently, Park et al. [32] made a significant contribution to the field by utilizing an SE-ResNet50 architecture on a large-scale nationwide dataset (22,522 deliveries) to validate, using retrospective data, the deep learning potential for detection of abnormal CTG, as assessed by the clinicians [32]. Their study establishes an important benchmark for data-driven generalization. Building on this progress, our work explores a complementary direction focused on model efficiency and clinical deployability. While Park et al. leveraged the high capacity of SE-ResNet50 ( 28 million parameters) to process extensive data, we investigated the feasibility of a lightweight alternative—EfficientNet-B0 combined with CBAM ( 5.00 million parameters, approx. 17.8% of the former’s size). Our findings suggest that even with a smaller dataset, a lightweight architecture enhanced by signal reconstruction and attention mechanisms can achieve competitive diagnostic performance. This implies that our approach could serve as a viable solution for resource-constrained clinical environments, offering a practical option alongside large-scale, high-complexity models.

**Figure 7:**
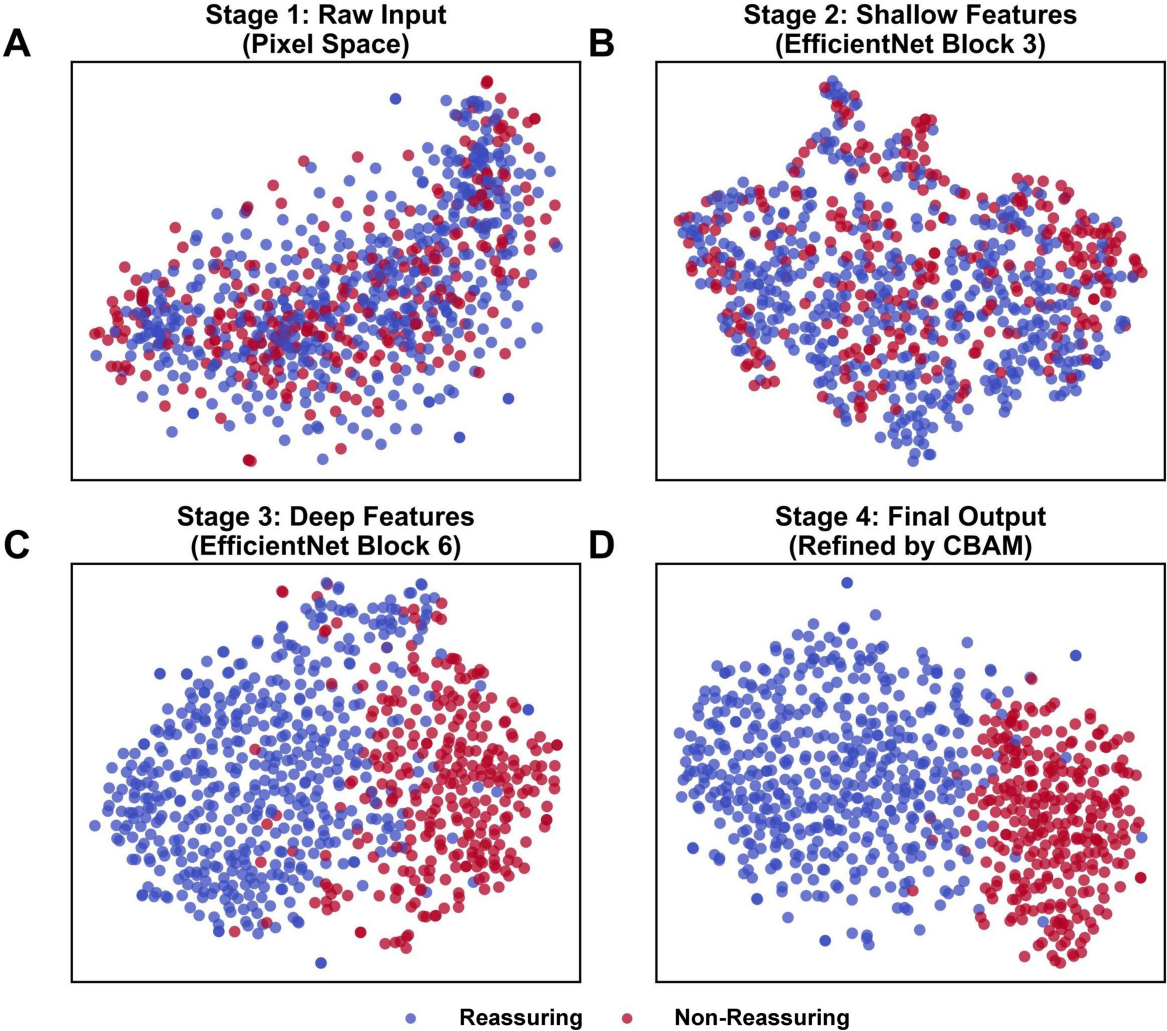
t-SNE visualization of feature distributions at different processing stages. The scatter plots illustrate the progressive evolution of feature separability between Reassuring (blue) and Non-Reassuring (red) samples. **(A)** Stage 1 (Raw Input): The original samples in pixel space are heavily entangled, showing no clear decision boundary. **(B)** Stage 2 (Shallow Features): Features extracted by the early layers (EfficientNet Block 3) begin to form local clusters but remain largely overlapping. **(C)** Stage 3 (Deep Features): Deeper layers (EfficientNet Block 6) extract more abstract semantic features, leading to the emergence of distinct group structures. **(D)** Stage 4 (Final Output): Features refined by the CBAM module show a clear inter-class separation and high intra-class compactness. This visual progression validates the model’s ability to transform complex raw CTG signals into discriminative diagnostic representations.

To further validate the discriminative capability of the proposed framework and provide insights into the feature extraction process of the deep learning model, we employed t-Distributed Stochastic Neighbor Embedding (t-SNE) to visualize the high-dimensional feature representations in a two-dimensional space. Figure 7 illustrates the progressive evolution of feature separability across four key stages of the network. Initially, in the raw input space (Figure 7A), the “Reassuring” and “Non-Reassuring” samples are largely overlapped, illustrating the complexity of the raw signal distribution. As the data propagates through the shallow (Figure 7B) and deep (Figure 7C) layers of the EfficientNet backbone, the model begins to extract increasingly abstract representations, though the decision boundaries remain indistinct. However, in the final output stage processed by the CBAM module (Figure 7D), the samples form more distinct clusters corresponding to the two classes. The distinct clusters formed in the final stage suggest that the CBAM module contributes to the separation of feature representations specific to fetal distress, providing visual support for the model’s approach.

## 4 Conclusion

The present study developed and validated a deep learning automated feature extraction method based on the EfficientNet-B0 architecture and a Convolutional Block Attention Module (CBAM) for the objective classification of fetal heart rate monitoring images. The workflow introduces a systematic image preprocessing pipeline designed to reduce background grid interference while maintaining signal integrity, coupled with a CBAM-driven mechanism that enables the model to adaptively focus on key discriminative features within FHR and UC curves. The model was trained on a private clinical dataset using clinician-labeled FIGO classifications (Normal vs. Suspicious/Abnormal) as the primary outcome. To evaluate its clinical utility and robustness, the model was externally validated on the public CTU-UHB dataset using objective umbilical artery pH levels (pH ≥7.05 vs. pH <7.05) as the benchmark. Experimental results demonstrate that the model achieves 92.66% accuracy on the internal test set and maintains a high accuracy of 95.65% on the CTU-UHB international public dataset. The performance of the proposed model is competitive with a range of baseline methods and conventional machine learning models.The performance observed across both visual and physiological benchmarks supports the potential utility of the proposed method in enhancing the automation and reliability of fetal status assessment.

Notwithstanding these achievements, several limitations were identified that also point to valuable future research directions:

1. The ensuing discourse will address the challenges associated with data scale and diversity. The training set utilised in this study was derived from a single centre, exhibiting comparatively limited sample size. This limit may affect the representation of variations across different regions, ethnicities, medical institutions, and acquisition devices. This could potentially influence generalization performance when the model is confronted with out-of-distribution data [33]. The establishment of a substantial, multi-centre fetal heart monitoring dataset represents a pivotal prerequisite for the advancement of this field [34, 35].
2. Potential impact of class imbalance: The skewed ratio of Reassuring or Non-Reassuring samples in the dataset (approximately 1.7:1) may cause model optimization to favour the majority class [36]. Despite contemporary models demonstrating acceptable recall and F1 scores for the abnormal category, the exploration of advanced loss functions or resampling techniques holds promise for further enhancing sensitivity in identifying rare yet clinically significant abnormal cases [37].
3. The clinical demand for interpretability of models is a subject that has attracted considerable attention of late [38]. The “black-box” nature of deep learning models presents a challenge to their clinical implementation. Subsequent endeavours will entail the incorporation of explainable AI methodologies for the purpose of visualising the decision-making processes of the model. The realisation of this objective is predicated on the rendering of the reasoning process transparent and traceable to clinicians. The establishment of clinical trust and the advancement of the practical implementation of diagnostic support systems is thus facilitated.

Consequently, future research intends to explore three pathways to further narrow the gap between efficient modeling and large-scale validation. Firstly, recognizing the importance of large-scale data for generalization, we plan to investigate federated learning-based collaborative paradigms. We hope this approach may allow for the aggregation of multi-center resources while respecting privacy protocols, potentially expanding the effective dataset size. Secondly, we aim to study advanced imbalanced learning algorithms combined with explainability frameworks. Our goal is to balance accuracy with interpretability, seeking to make the model’s attention to morphological features clearer to clinicians. Finally, we aspire to conduct prospective clinical trials to further assess the system’s reliability in real-world scenarios. By extending validation to larger, multi-center cohorts, we hope to better evaluate the lightweight model’s potential utility as a clinical decision support tool.

## Data Availability

The datasets presented in this article are not readily available because The data involves patient privacy, and a data confidentiality agreement has been signed with the hospital. Requests to access the datasets should be directed to Xinghe Zhou, 1021623364@qq.com.
