# Correction: Objective interpretation of intrapartum cardiotocography images using attention-guided convolutional neural networks

**DOI:** 10.3389/fped.2026.1846046

**Published:** 2026-05-19

**Authors:** Xinghe Zhou, Tianxin Qiu, Chunxia Lin, Jun Zhou, Shiling Jiang, Litao Wang, Li Feng, Xinhao Wang, Qingshan You

**Affiliations:** 1 Faculty of Science, Civil Aviation Flight University of China, Chengdu, Sichuan, China; 2 Department of Obstetrics, West China Longquan Hospital Sichuan University, The First People’s Hospital of Longquanyi District Chengdu, Chengdu, Sichuan, China

**Keywords:** CBAM, computer vision, deep learning, efficient net, electronic fetal monitoring, fetal status classification, intrapartum monitoring

The affiliations were erroneously given as ^1^Department of Obstetrics, West China Longquan Hospital Sichuan University, The First People's Hospital of Longquanyi District Chengdu, Chengdu, Sichuan, China; ^2^Faculty of Science, Civil Aviation Flight University of China, Chengdu, Sichuan, China.

The correct affiliations are as follows.

^1^Faculty of Science, Civil Aviation Flight University of China, Chengdu, Sichuan, China; ^2^Department of Obstetrics, West China Longquan Hospital Sichuan University, The First People's Hospital of Longquanyi District Chengdu, Chengdu, Sichuan, China

Corresponding author

Author Chunxia Lin was erroneously missed being assigned as corresponding author. The correct corresponding authors are Chunxia Lin, 986138020@qq.com and Qingshan You, youqingshan@cafuc.edu.cn.

The original version of this article has been updated.

